# Spawning Asynchrony and Mixed Reproductive Strategies in a Common Mass Spawning Coral

**DOI:** 10.1002/ece3.71654

**Published:** 2025-06-26

**Authors:** Gerard F. Ricardo, Christopher Doropoulos, Russell C. Babcock, Arne A. S. Adam, Elizabeth Buccheri, Natalia Robledo, Julian Uribe‐Palomino, Peter J. Mumby

**Affiliations:** ^1^ Marine Spatial Ecology Lab School of the Environment, The University of Queensland St. Lucia Queensland Australia; ^2^ CSIRO Environment St. Lucia Queensland Australia

## Abstract

Understanding strategies of organisms that utilise multiple modes of reproduction presents a complex challenge for evolutionary biologists. The genus *Platygyra*, a common reef‐building coral with unclear reproductive boundaries among morphological species, illustrates these complexities. Here, we evaluate the contribution of different reproductive modes in the coral *P. daedalea* at Heron Island, on the southern Great Barrier Reef, during the less prominent phase of a split‐spawning event, enabling assessment of reproductive outcomes associated with low population densities. We tagged and sequenced eighteen coral colonies, representing varying degrees of spatial clustering, along a 130‐m stretch of shallow reef slope. During spawning, divers trapped eggs from monitored colonies in mesh containers, which were then released into the surrounding water. High levels of spawning asynchrony were observed, potentially indicating distinct genetic clusters within the putative species, resulting in low fertilisation success (1.5%). Notably, paternity assignments revealed all resulting embryos were self‐fertilised, with no cross‐fertilisation detected, suggesting that self‐fertilisation may serve as a reproductive assurance mechanism in *Platygyra* during these smaller spawning events. The adult population showed evidence of two genetically distinct subpopulations, along with spatial autocorrelation and inbreeding. This supports the existence of smaller breeding units within larger assemblages, density‐dependent population effects and localised recruitment, indicating that these populations may be more hierarchically structured than previously recognised. Given the lack of evidence for in situ outcrossed fertilisation in this natural coral population during a minor spawning event, it appears that *P. daedalea* may utilise atypical modes of reproduction at low densities as observed here and/or alternatively rely on higher‐density spawning events and favourable hydrodynamic features not captured in this study.

## Graphical abstract

The coral *Platygyra daedalea* exhibits complex reproductive strategies, with individuals spawning asynchronously during a split‐spawning event and showing limited cross‐fertilisation. Genetic analysis suggests small breeding groups within larger populations, leading to low fertilisation success and reliance on self‐fertilisation. These findings highlight the influence of population density and spawning asynchrony on fertilisation success and coral reproduction.

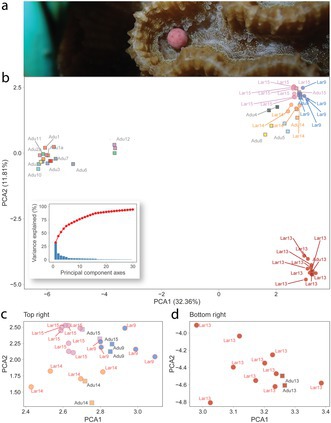

## 1 Introduction

Colonial organisms, particularly sessile species, face unique challenges related to reproduction, given their spatial limitations and the need for gamete dispersal. To overcome these challenges, many colonial organisms employ multiple reproductive strategies, such as external fertilisation and a mix of sexual and asexual reproduction (Heyward and Babcock 1986; Ayre and Hughes 2000; Yang and Kim 2016). Similar to higher plants, colonial invertebrates often share many characteristics, including sessile adult stages, hermaphroditism and synchronous external reproductive events that produce masses of progeny (termed ‘mast seeding’ in plants (Kelly 1994) and ‘mass spawning’ in benthic invertebrates (Harrison et al. 1984)). Both groups face unique challenges related to mating including how to achieve fertilisation and exercise mate‐choice or optimise sex allocation (Serrão and Havenhand 2009). One explanation for the persistence of colonial organisms is their use of multiple reproductive strategies over evolutionary and generational timescales, or in response to different environmental conditions (Williams 1975; Barrett 2014; Uecker 2017).

Outcrossed sexual reproduction, or outcrossing, is the fusion of gametes from different individuals. Outcrossing can enhance genetic diversity and tolerance to environmental disturbances but often requires higher energetic investment owing to meiosis and, in externally fertilising species, gametic loss (Hartl 1997). In broadcast‐spawning invertebrates, increased distances between individuals and the dilution of gametes during spawning can reduce the likelihood of gamete encounters, leading to lower reproductive success, i.e., a component Allee effect (Levitan and Petersen 1995; Courchamp et al. 1999). Similarly, in higher plants, at least 10% of angiosperms rely on wind pollination, a process that carries a similar risk of low fertilisation from pollen limitation (Friedman and Barrett 2009). In these plants, critical distances for reduced fertilisation can exceed 60 m (Knapp et al. 2001). In contrast, the threshold for reduced fertilisation in sessile invertebrates is less well understood and may be much shorter, potentially restricted to just tens of metres (Coma and Lasker 1997; Mumby et al. 2024). Although reproductive processes in terrestrial systems, particularly higher plants, have been extensively studied, our understanding of reproductive isolation and the dynamics of mixed reproductive modes in sessile colonial invertebrates remains limited, highlighting a critical knowledge gap.

Self‐fertilisation, or selfing—a mode of sexual reproduction between a single individual's gametes—is common in hermaphroditic organisms (Escobar et al. 2011). Approximately 15%–20% of hermaphroditic animals, including many sessile species, are highly selfing (Jarne and Auld 2006). Similarly, around 10%–15% of flowering plants, a major group of higher plants, predominantly self‐fertilise (Wright et al. 2013). Selfing provides reproductive assurance in the absence of mates, helping to maintain adaptive genes suited to local conditions (Dornier et al. 2008). Over evolutionary timescales, shifts from outcrossed to selfing‐dominated reproductive modes are common (Wright et al. 2013). However, selfing lineages tend to be short‐lived, as these lineages eventually face challenges associated with low genetic diversity, such as inbreeding depression and reduced capacity to respond to environmental disturbances (Goodwillie and Weber 2018). Likewise, asexual reproduction also enables rapid local expansion, but the reliance on clonal propagation can reduce genetic diversity, increasing vulnerability to environmental stress (but see Kirkpatrick and Jarne (2000)). Species that rely on asexual modes, typical of colonising species, are subject to frequent reductions in population densities (Manning 1981), but nevertheless can be successful. In some cases, asexual clones can reach staggering sizes: a polyploid clone of seagrass in Western Australia has reached 180 km and survived for 8500 years (Edgeloe et al. 2022).

Different modes of reproduction generate varying levels of genetic diversity through distinct biological processes. Outcrossed sexual reproduction increases genetic diversity via meiosis and the fusion of chromosomes from two parents. Selfing also permits some genetic diversity through the processes of recombination and independent assortment of chromosomes during meiosis, but it is more limited because both gametes originate from the same individual (Hartl 1997). Although selfing rate estimates are often inferred from indirect population‐level metrics, these metrics can be heavily biased if assumptions about population structure, inbreeding or genotyping accuracy are violated (Bürkli et al. 2017). Data from progeny arrays using genetic markers provide more direct estimates of selfing with fewer assumptions compared to population‐level metrics. However, progeny arrays are rarely conducted in animals, especially in situ, because of their cost‐ and labour‐intensive nature (Hartl 1997; Jarne and Auld 2006). Examples in sessile colonial invertebrates are limited, though a few notable studies exist (Coffroth and Lasker 1998; Lasker et al. 2008; Warner et al. 2016; Dubé et al. 2020). Asexual reproduction, such as fragmentation, typically results in progeny with the same genomic arrangement as the parent, apart from rare mutations. Parthenogenesis, a type of asexual reproduction where an egg develops into an embryo without fertilisation, is widespread in terrestrial taxa including plants, insects and reptiles (Bierzychudek 1985; Watts et al. 2006; Normark and Kirkendall 2009), but is less well understood in sessile colonial invertebrates (Ayre and Miller 2004; Levitan et al. 2025). Parthenogenesis can arise through mitotic or meiotic mechanisms, resulting in either no or limited genetic variation.

Mixed reproductive modes have been extensively studied in higher plants, but sessile colonial invertebrates such as corals offer valuable yet underutilised model organisms to examine these evolutionary roles. Their value stems from their diverse reproductive strategies, sessile nature and the general interest in how these taxa may adapt under broad‐scale disturbances such as climate change. Approximately 80% of corals release gametes into the water column (Harrison 2011), allowing for random mating and dispersal. Brooding, which occurs in about 17% of coral species and can involve either sexual or asexual reproduction, is a less common reproductive strategy (Whitaker 2006; Baird et al. 2009). Although selfing is generally considered uncommon in corals, there are a few notable genera that exhibit high levels of selfing, such as *Goniastrea*, *Favia*, *Seriatopora*, *Porites* and *Platygyra* (Willis et al. 1997; Gleason et al. 2001; Miller and Mundy 2005). Various modes of asexual reproduction that result in clonal propagation exist in corals, such as fragmentation of various life history stages (Wallace 1985; Babcock 1991; Heyward and Negri 2012), polyp‐bailout (Sammarco 1982) and parthenogenesis (Combosch and Vollmer 2013; Vollmer 2018).

Previous studies have documented a range of in situ fertilisation outcomes influenced by spatial population metrics, species traits and hydrodynamic factors (Coma and Lasker 1997; Levitan et al. 2004; Miller and Mundy 2005; Mumby et al. 2024; Ricardo et al. 2024). However, it remains unknown whether successful fertilisation can occur during minor spawning events, where altered gravid population densities may influence gamete concentrations and fertilisation success. Split spawning—where individuals at a given location release gametes across multiple months—provides a unique natural experiment to examine shifts in reproductive strategies under variable environmental conditions. Although split spawning has been hypothesised to realign reproduction with favourable conditions (Willis et al. 1985; Foster et al. 2018), these separated spawning events may also elevate the risk of fertilisation failure, particularly in low‐density populations, by further reducing the effective pool of gametes. Assessing these low‐density spawning populations may also provide insights into how degraded populations might respond under ongoing threats such as climate change. In this study, we use the coral *Platygyra daedalea* as a model to investigate mixed modes of reproduction during a smaller fraction of a split spawning event. Although selfing occurs in *Platygyra* at elevated levels relative to other coral genera, outcrossing remains the primary mode of reproduction (Willis et al. 1997). To investigate these reproductive modes, we employ next‐generation sequencing for paternity assignments and population structure analysis to directly assess the relative contributions of outcrossing, selfing and asexual reproduction. Specifically, we address three key questions: (1) What is the effect of adult colony spacing and density on outcrossed fertilisation success? (2) Is there a role of self‐fertilisation in overall fertilisation outcomes? (3) Can we detect any potential presence of asexual reproduction within our coral patch?

## 2 Methods

### 2.1 Coral and Site Selection

**FIGURE 1:**
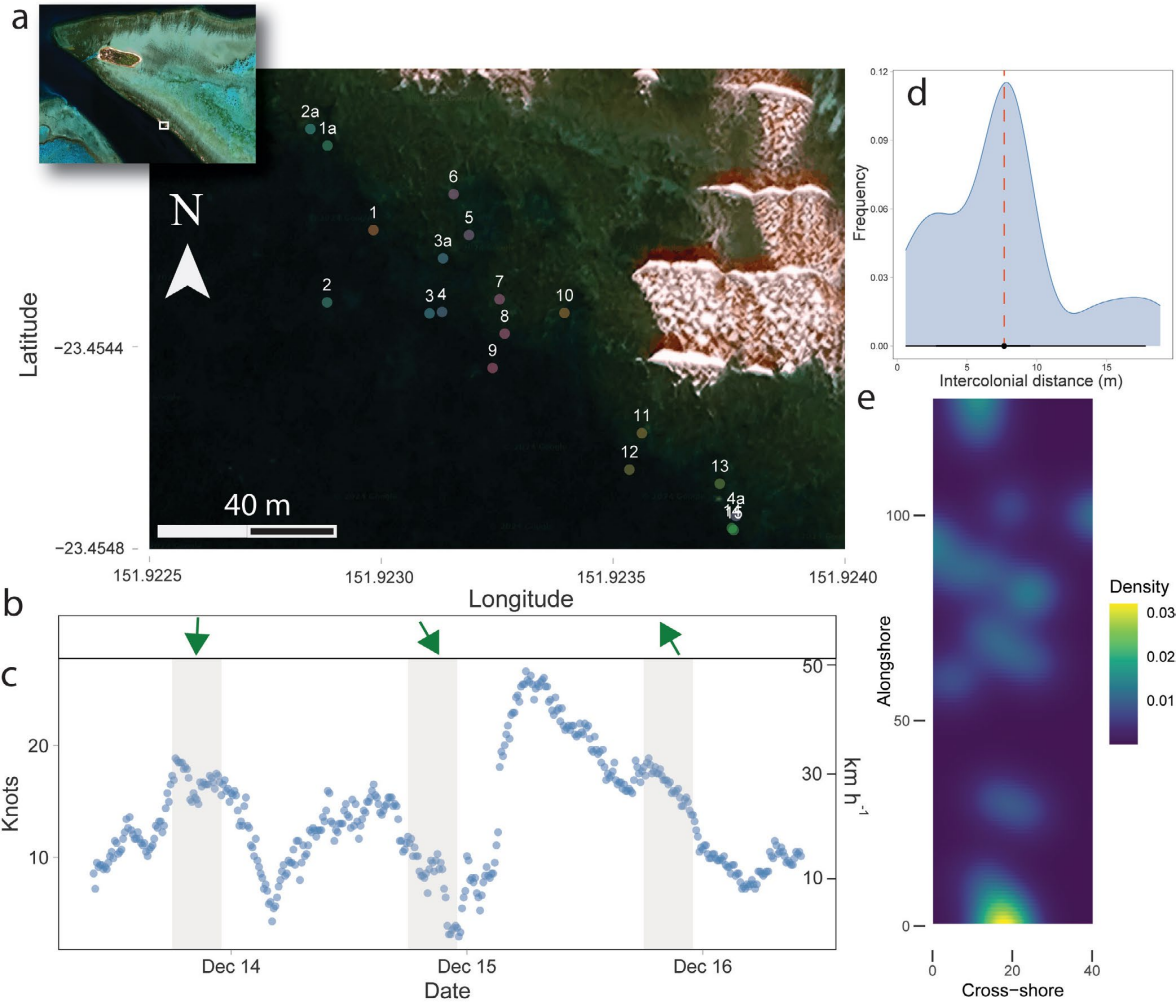
Patch and environmental conditions during the spawning period. (a) Patch distribution of gravid adult *Platygyra daedalea* colonies along the reef slope (2–9 m depth). (Inset: The western side of Heron Reef and field site.) (b) Wind direction (green arrows) and (c) wind speed during the spawning period. Grey boxes denote nightly spawning periods. (d) Intercolonial distance and spatial distribution densities of gravid colonies within the patch. The red line indicates median intercolonial distance. The low bar indicates the 66% (thick) and 95% (thin) probability intervals. (e) Density of gravid colonies within the patch showing the degree of spatial clustering.

Our chosen field site was situated at Coral Canyons (23.4548° S, 151.9238° E), located on the western side of Heron Reef, adjacent to the Heron‐Wistari channel. This location is marked by distinctive tidal currents along the reef slope, flowing northwest during flood tides and southeast during ebb tides, and confirmed with the release of drogues on both tide cycles. Wind speed and direction data were extracted from the Australian Institute of Marine Science (AIMS) Heron Island weather and oceanographic Station dataset (Barneche et al. 2021), and confirmed in situ with a handheld anemometer. The site is exposed to waves during common south‐easterly swells, median peak period of 4.7 s and a median significant wave height of 0.76 m (Callaghan 2023; Figure S1), and featured spur‐and‐groove formations—alternating ridges (spurs) and channels (grooves) shaped by wave action throughout the site (Figure 1a). Such formations are common on wave‐exposed fore reefs throughout the world (Rogers et al. 2013).

The genus *Platygyra* is known as a syngameon, with unclear reproductive boundaries between morphological species (Miller and Babcock 1997). Among these, *Platygyra daedalea* is primarily a hermaphroditic submassive coral that usually spawns six to eight days after the full moon on the southern GBR (Baird et al. 2021). Egg‐sperm bundle setting can be difficult to observe, and the species is known to have spawning times ranging from dusk until several hours after sunset (Miller and Babcock 1997). At our location, *Platygyra* species are known to have their main spawning event in November in a regular (non‐split) spawning year (Eyre et al. 2008; Baird et al. 2021; Doropoulos and Roff 2022). The colony releases egg‐sperm bundles instantaneously within just a few minutes, and egg‐sperm bundles contain ~60 eggs per bundle, with eggs being 300–400 μm in diameter (Babcock et al. 2003; Baird et al. 2015). Successfully fertilised eggs of this genus undergo initial cleavage at 1–2 h post fertilisation (Babcock and Heyward 1986; Oliver and Babcock 1992).

Corals of *P. daedalea* were examined for the presence of pigmented eggs starting two days prior to the full moon on the 8th of December 2022. As corals on the mid‐shelf and outer Great Barrier Reef typically spawn in November, a split spawning was predicted owing to the early full moon occurring in December (Baird et al. 2009), increasing the risk of Allee effects occurring during fertilisation. A preliminary survey revealed a very low proportion of gravid corals in most genera sampled, with approximately 25% of the *P. daedalea* population on the reef slope showing mature oocytes indicating a minor spawning event. We did not observe any colonies with immature eggs suggesting that the major spawning event had occurred after the previous full moon. All corals of *P. daedalea* along 150 × 36 m of the reef slope were assessed for gravidity by progressively shearing small layers of tissue off the coral's surface to expose the egg layer. Only gravid colonies from the study site with a mean diameter of ≥ 10 cm were included, and a total of 18 gravid corals were tagged along the reef slope, which extended from ~2 to 9 m depth, with 15 monitored during spawning periods. Most morphotypes were identified as *P. daedalea* PDC (classic morph) following descriptions by Miller (1994a). *Platygyra* species are known to create hybrid embryos, but only one other species was found in the patch ( *Platygyra sinensis* ), and this individual was not gravid. During daylight hours, each tagged colony was georeferenced using a handheld GNSS receiver (Garmin eTrex 10, horizontal accuracy < 5 m). Colony size was measured by taking an image of the colony from directly above and using a graduated diving rod as a scale (mean ± SD planar diameter = 24.0 ± 11.4). A marking line was run sequentially between each gravid colony, enabling divers to quickly identify any indications of spawning (see Experimental Design Section 2.2 below).

### 2.2 Experimental Design

To assess in situ fertilisation success across the transect, we constructed spawn collectors by mounting a funnel above the coral using metal wire frame. The funnel channelled gamete bundles into a detachable Falcon tube (herein ‘mesh containers’). A portion of the container had been removed and covered with 150‐μm mesh to allow for sperm to move freely through the containers while retaining the individual colony's eggs. Glowsticks were securely fastened to the mesh containers for easy tracking during the night. The mesh containers were positively buoyant, with approximately three‐quarters of the container remaining submerged once on the water's surface. Movement of sperm through the mesh to fertilise eggs has been validated by Ricardo et al. (2024), confirming the effectiveness of this approach.

During the predicted coral spawning window (11–16 December), a team of four divers was deployed at sunset (~18:00), and corals were monitored along the marking line using red‐beam dive torches. Spawning was observed from 13 to 15 December. When spawning was observed in individual colonies, the mesh containers were immediately removed from the collector, capped, and their attached glowsticks were activated before being released into the water column. Released mesh containers were tracked by vessel for over 80 min, with their collection locations marked using a GNSS receiver. After retrieval, the mesh containers were transferred to individual containers filled with sperm‐free seawater collected prior to spawning and transported to Heron Island Research Station for assessment. In addition to the experimental mesh containers, three in situ self‐fertilisation controls were collected by retaining spawned egg‐sperm bundles in closed and sealed containers. Gametes within each mesh container were scored at 3 h post‐spawning, corresponding to the onset of the 4‐cell stage, with fertilisation success recorded as the proportion of embryos relative to the total initial eggs (*n* > 200 per container). Gametes were continuously assessed alongside the ex situ selfing experiment described below until approximately 6 h post spawning. Embryos from containers were then isolated into individual wells of 6‐well plates containing filtered seawater for development into 36‐h‐old larvae for later genetic work. Embryos were isolated at ~4‐cell stage, before they became fragile, thereby preventing the risk of fragmentation (Ricardo et al. 2016). In addition to the released mesh containers, bundles from two colonies were captured in closed containers to assess self‐fertilisation and processed as above.

In addition to the main field experiment, other modes of reproduction were assessed *ex situ* to assess the maximum role of self‐fertilisation and also parthenogenesis. To assess self‐fertilisation, two colonies outside of the patch were collected and brought back to aquaria facilities at Heron Island Research Station. A single colony spawned on the 14th of December 2022 and was used as a self‐fertilisation control. The eggs were left in ~10^7^ sperm mL^−1^ and assessed at three and six hours following spawning. During the following year, parthenogenesis was assessed by collecting egg‐sperm bundles from two isolated colonies of *P. daedalea* . Bundles were separated into eggs and sperm using a 100‐μm mesh. Eggs were washed with filtered seawater and divided into containers (*n* = 3 per individual) containing 100 mL of filtered seawater. The eggs were assessed the following day for indications of fertilisation via observations of embryo development.

### 2.3 Genetics Sampling and Analyses

Genetic sequencing was used for paternity assignments and to assess population structure. A small amount of tissue from each of the monitored 15 colonies, and an additional three unmonitored colonies, was collected into zip‐lock bags for sequencing. Replicate samples (*n* = 2) of adult coral colony tissue were stored in 100% ethanol at −80°C until further processing in the lab. Embryos underwent development for more than 36 h before being transferred in 10‐μL seawater into 200‐μL of 100% ethanol solution. The samples were transported to the University of Queensland and stored at −20°C. *Prior* to sequencing, samples of adults and larvae were transferred to 96‐well plates (Eppendorf, TwinTec, skirted, 150 μL, PCR clean), capped (Eppendorf, Cap Strips, 8‐strips, domed) and then sent to Diversity Arrays Technology (DArTSeq; Canberra, Australia) for DNA extraction, library preparation, sequencing and single nucleotide polymorphisms (SNP) identification. DArTSeq sequencing is a technique that employs pairs of restriction enzymes to digest genomic DNA, enabling the identification of genome‐wide SNP markers. Adapters are added to the sequence fragments to facilitate sequencing on an Illumina short‐read platform. Sequencing was carried out on the Illumina platform, producing raw “sequence tags” of approximately 75 base pairs. These terminal sequences include a barcode to allow disaggregation of sequences from each sample during subsequent analysis. The sequence tags are filtered on the basis of sequence quality, especially in the barcode region, and truncated to 69 base pairs. The tags are then grouped by sequence similarity. A series of proprietary filters are applied to select reliable SNP markers. One‐third of the samples are processed twice as technical replicates, using independent adaptors, through to allelic calls. High‐quality markers with low error rates are primarily selected using scoring consistency (repeatability). Sequencing was conducted against a reference *P. daedalea* genome v1.0 (Voolstra et al. 2015; Liew et al. 2020). Codominant, genome‐wide, biallelic SNP markers were detected at 0.8 million reads.

Raw SNP sequencing data were subjected to standard filtering processes. Loci sharing SNPs were identified and duplicates removed, retaining only one fragment per set. Next, loci were filtered to a minimum read depth of 10. Loci with limited reproducibility were also excluded using a threshold of 95%. Additionally, loci and individuals with low call rates, indicating high proportions of missing data, were filtered out using thresholds of 70% and 58% respectively. SNP markers were filtered by retaining loci where the SNP and reference allele were between 0.01 and 0.99 and where sequencing coverage exceeded 4 reads to ensure the inclusion of polymorphic loci with sufficient depth.

### 2.4 Paternity Assignments and Progeny Arrays

Paternity assignments were conducted on offspring found within each mesh container. Colonies that contributed eggs and were tracked within mesh containers were hereafter referred to as ‘egg donors’, whereas initially unidentified colonies that provided sperm for fertilisation are hereafter referred to as ‘sperm donors’. As a proof of concept, we also tested if paternity assignments of larvae (*n* = 3) from known parents could be determined correctly from a pool of candidate parents (*n* = 11). Full details of this validation experiment are provided in Text S1. For the main experiment, samples of each colony (*n* = 2) and 42‐h‐old larvae originally extracted from the mesh containers were placed in 100% ethanol solution and stored at −20°C. These samples were sent for SNP sequencing (0.8 mln reads) and basic filtering at DarT P/L as described above. SNP data underwent further filtering that reduced the total number of usable loci to 644, which is typically sufficient to predict parent‐offspring pairs in SNP data (Anderson and Garza 2006; Premachandra et al. 2019).

Paternity assignments were carried out using the CERVUS 3.0.7 software (Kalinowski et al. 2007). CERVUS is a simulation‐based approach that uses likelihood ratios to assign parentage. The simulation‐based approach is relatively robust to null alleles and genotyping errors (Kalinowski et al. 2007). The analysis was under strict (95%) confidence levels for assignments. To assign the unknown sire (sperm donor colonies), the following settings were used: simulated offspring = 10,000, candidate sires (sperm donors) = 19, proportion of colonies sampled = 0.8, proportion of loci typed = 0.95, proportion of loci mistyped = 0.05, minimum typed loci = 500. An 80% sampled candidate sperm donor's rate was selected because of a small proportion of unsampled colonies that are found on the reef flat upstream of the site.

CERVUS cannot identify unsampled candidate parents, but the uncertainty in the simulations is reflected in the level of confidence in the assignments. To increase robustness and certainty, the paternity assignment was repeated using the software COLONY 2.0 (Jones and Wang 2010), using the following inputs: Mating System I = polygamy, Mating System II = inbreeding, clone, Species = monoecious, Analysis Method = Full‐Likelihood, with the probability of sperm donors within the patch having equal probability. Further, changes in heterozygosity between known parents and offspring would additionally support selfing (Ayre and Miller 2006) and were compared using $F_{\text{IS}}=1-H_{o}/H_{e}$, where $H_{o}$ is the observed heterozygosity and $H_{e}$ is the expected heterozygosity. $F_{\text{IS}}$ between parents and offspring were compared using a *t*‐test after checking for normality and homogeneity of variances. Genetic variation of the larvae in respect to the adults were visualised using Principal Component Analysis (PCA) with the package *ade4* (Dray and Dufour 2007).

### 2.5 Subpopulation Structure

Null alleles can falsely increase homozygosity (David et al. 2007), and an additional filtering step was added using the package *popgenreport* (Adamack and Gruber 2014) for analyses more sensitive to these artefacts. To assess the genetic structure across the adult *P. daedalea* population at our site, we performed Principal Component Analysis (PCA) ordination, combined with density‐based spatial clustering DBSCAN (Hahsler et al. 2019), or model‐based clustering methods using STRUCTURE (Pritchard et al. 2000). For the PCA, the first two principal components were used to assess genetic groups. Appropriate eps values for DBSCAN clustering were determined using k‐nearest neighbour distance plots, with the elbow method to identify the optimal threshold. For STRUCTURE analysis, the Evanno method was used to determine the optimal *K* value for genetic groups using the admixture model. For both analyses, only the highest quality replicate of each unique individual was analysed to prevent artificial clustering. Similarly, the inbreeding coefficient (*F*), defined as the probability for an individual to inherit two identical alleles from a single ancestor, was calculated with the package *adegenet* (Jombart and Ahmed 2011).

Linkage disequilibrium was assessed using the Index of Association (IA) using the package *poppr* (Agapow and Burt 2001; Kamvar et al. 2014). IA indicates the non‐random association of alleles between loci. During sexual fertilisation, meiosis shuffles alleles through recombination, leading to random associations of alleles. Therefore, non‐random association can indicate the absence of recombination, as seen in mitotic mechanisms such as clonality, but may also indicate subpopulation structure.

### 2.6 Statistical Analysis

Georeferenced individuals were analysed for nearest‐neighbour distances and Clarke‐Evans index using the package *spatstat* (Baddeley and Turner 2005) in R (version 4.2.1). A Mantel test was conducted to assess pairwise differences in spawning times between individuals and their pairwise distances using the package *vegan* (Dixon 2003). A Fisher exact test was used to compare genetic groups with spawning times. Spawning time was binned into categories ‘early’ and ‘late’ using a 45‐min time threshold because exact spawning times were not available for corals that spawned after the divers had exited the water. A two‐sample *t*‐test was used to compare the main genetic groups (categorical) with depth (continuous).

## 3 Results

### 3.1 Environmental Conditions

During the two primary spawning days that the experiment was conducted, winds from the north to northwest at 22 ± 7 km h^−1^ (12 ± 4 knots) were observed, typical for this region during spawning (Barneche et al. 2021) (Figure 1). Tides occurred during the low or early flood stages at the time of spawning, transitioning to a flood tide during the periods of mesh container release (Table 1). Although low to moderate winds prevailed from the N to NW direction, the movement of the spawn and mesh containers was primarily driven by tidal currents in a W–NW direction (Figure S2b). The mesh containers moved at a speed of 0.05 m s^−1^ on both nights (approximately 270 m during the 90‐min release). On the second night of spawning, the final positions of the mesh containers were aligned parallel to the reef at 161.2 m ± 10.3 from shore, despite different release times. This may indicate a topographically controlled front which forms commonly on the western sides of Heron Reef, noticeable by the long algal blooms of *Trichodesmium* observed along the reef during daylight hours and coral spawn slicks on the days following spawning events (Figure S3a). Release of drogues near the site on flood and ebb tides indicated that tidal currents ran parallel to the reef slope (Figure S3c).

**TABLE 1:** Meteorological conditions during the nights of the experiment (Barneche et al. 2021).

Spawning date	Predicted tidal height (m)	Tide direction	Tide amplitude (m)	Wind speed (km/h, [knots])	Wind direction (°)	Last light
13 December	1.2	Early flood	1.4	26 (31), [14 (17)]^a^	N (6)	18:58
14 December	1.3	Slack low	1.3	13 (17), [7 (9)]^a^	NW (333)	18:59
15 December	1.2	Slack low	1.3	28 (31), [15 (17)]^a^	SSE (165)	18:59

^a^ Parentheses denote maximum wind speed.

### 3.2 Patch Characteristics

Of the gravid *P. daedalea* colonies along the transect, the median intercolonial distance was 7.7 m, and the density was 0.004 gravid individuals m^−2^. The Clarke‐Evans index was 0.784, indicating spatial clustering (Figure 1e). After considering genetic groupings (see below), Group 1 resulted in a median intercolonial distance of 14.4 m and a density of 0.002 gravid individuals m^−2^, whereas Group 2 resulted in a median intercolonial distance of 7.99 m and a density of 0.003 gravid individuals m^−2^. Corals within the transect spawned asynchronously across days and time from last light (Table S1). Twelve of the fifteen colonies showed visible evidence of spawning during the monitoring period, and the remaining three colonies were missing eggs upon reassessment.

Although there was a strong positive relationship between spawning times of individuals and their intercolonial distances on the first spawning night, the relationship was not statistically significant (*r* = 0.993, *p* = 0.250, *n* = 4), likely owing to the low statistical power owing to few replicates. The second night of spawning showed no significant relationship between spawning times and intercolonial distance (*r* = 0.112, *p* = 0.186, *n* = 6). When genetic groups were also assessed for spawning times using ‘early’ or ‘late’ binning, all individuals in Group 1 spawned late (i.e., > 45 min after sunset), whereas five of seven individuals in Group 2 spawned early (*p* = 0.061).

### 3.3 Fertilisation Success

**FIGURE 2:**
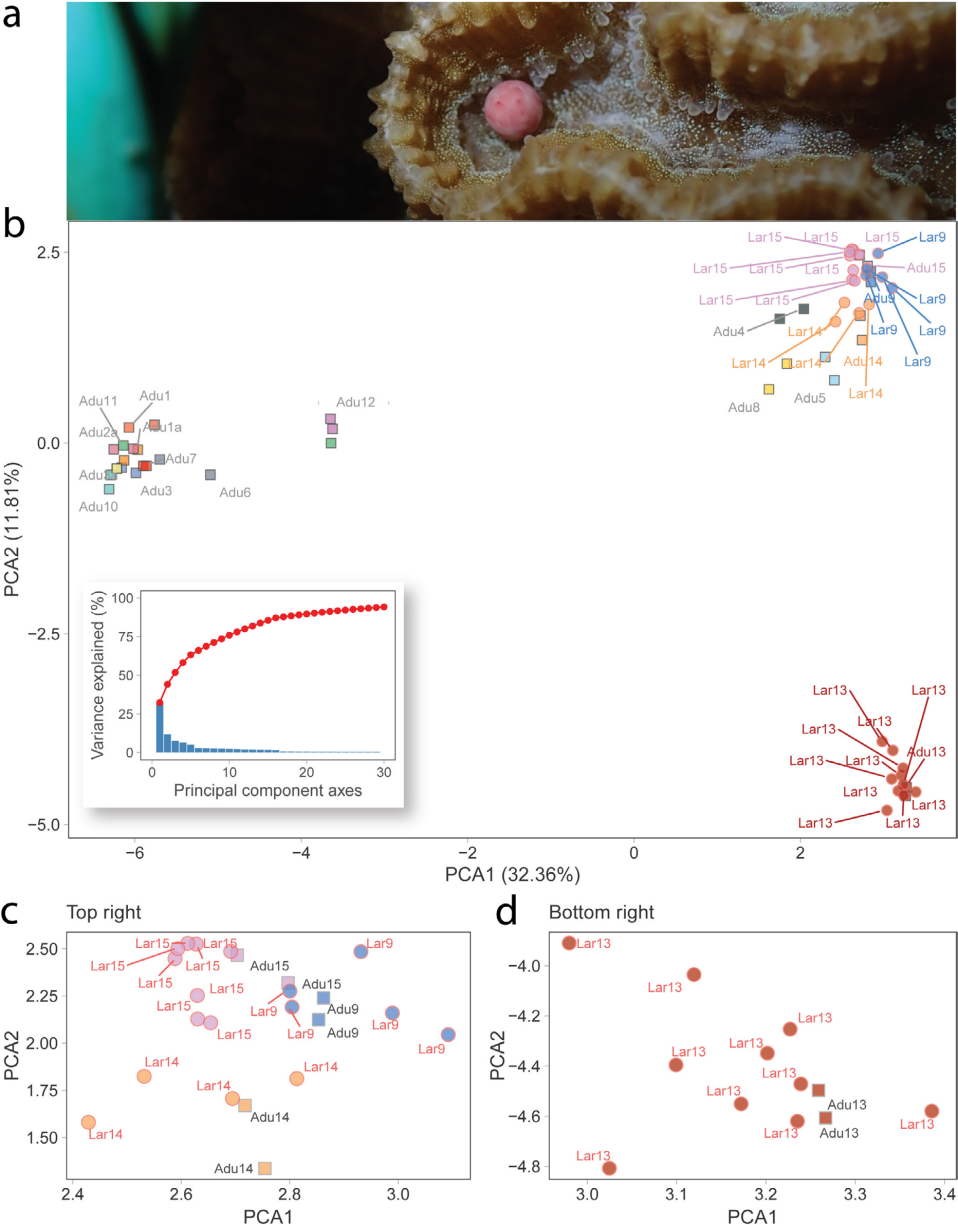
(a) *Platygyra daedalea* releasing an egg‐sperm bundle during spawning. (b) Principal Component Analysis (PCA) of adults and larvae. Square borders denote adults, circle borders denote larvae. Each genotype is coloured individually, and larval colouring matches that of known egg‐donor colony genotype. Labelling corresponds to colony ID number (adults) or egg‐donor colony ID number (larvae). Only parental genotype labels are coloured. (Inset) Variance explained by each principal component axis. (c) A subset plot of the top‐right cluster and (d) a subset plot of the bottom‐right cluster. Labels for adults are coloured grey and for larvae coloured red.

Owing to many corals spawning outside of the diver monitoring periods, combined with the loss of two mesh containers, only five containers were successfully released and collected. The mean fertilisation success in the experimental samples was 1.5%. Although self‐fertilisation in the field control samples (*n* = 3) was 0%, all individuals that were successfully sequenced (*n* = 28) were identified as selfed embryos via paternity assignments under both CERVUS and COLONY analyses, closely matching the genetic characteristics of their known egg donor (Tables S2, S3 and Figure 2). Further, there was a relatively significant decrease in heterozygosity as indicated using $F_{\text{IS}}$ between known parents and offspring (*t* = −6.3858, df = 6, *p* < 0.001).

Further, the laboratory control for selfing, where eggs from a single individual were exposed to its own sperm at > 10^6^ sperm mL^−1^ for an extended contact time, resulted in low self‐fertilisation (< 1%) at 3 h post‐spawning. However, at 6 h, the controls were again assessed and found to be 15% fertilised. All the selfed embryos at this census time point were in the two‐cell stage, indicating self‐fertilisation was delayed. Contamination of sperm from other individuals was not possible, as only one colony spawned ex situ on that night. There was no evidence of parthenogenesis in the eggs washed free of sperm.

### 3.4 Subpopulation Structure

**FIGURE 3:**
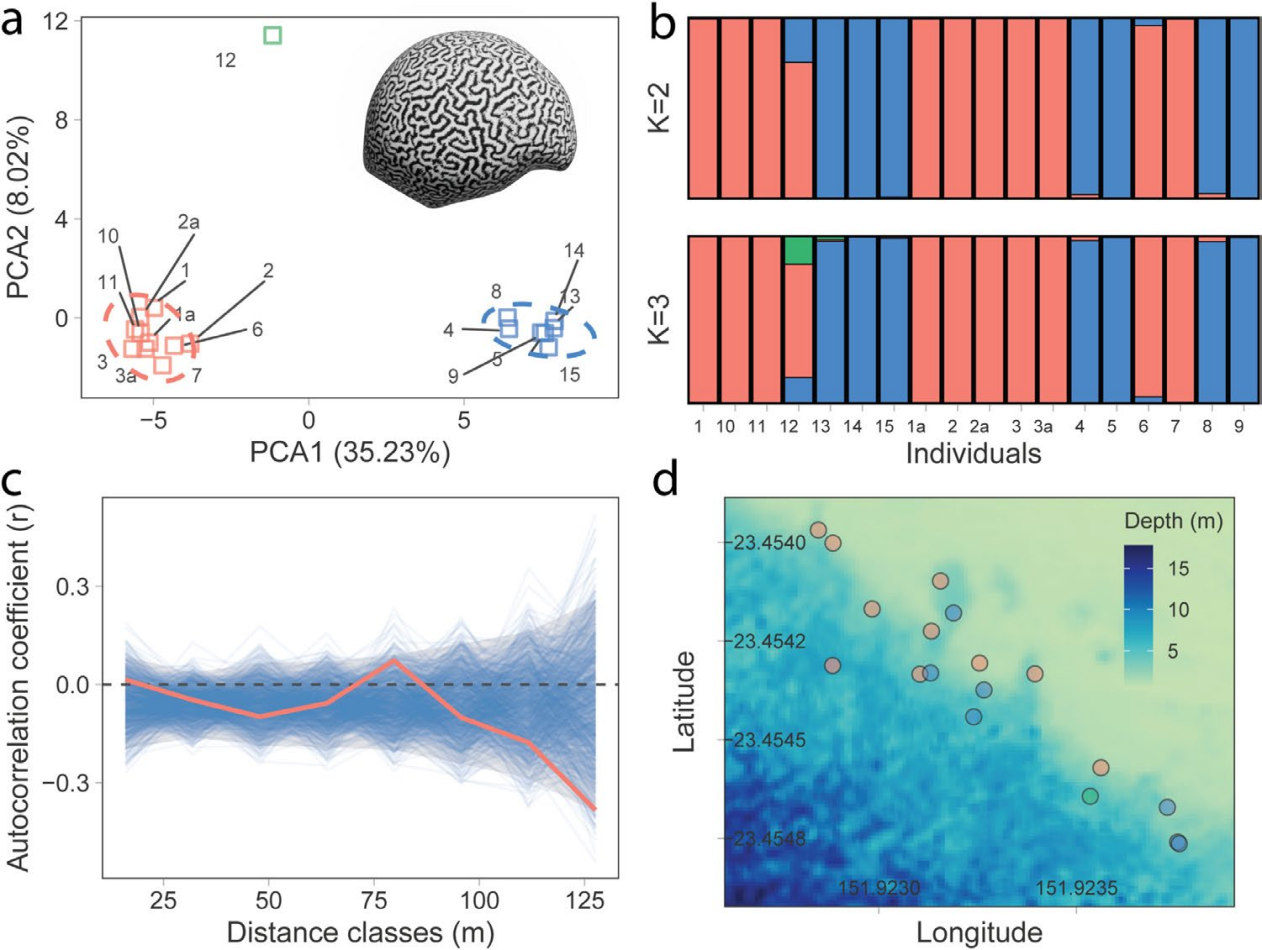
Genetic clustering and spatial relationships among the adult *Platygyra daedalea* colonies within the transect. (a) Principal Component Analysis (PCA) ordination of unique adult colonies. Group 1 = blue. Group 2 = red. Group 3 = Green. (b) Admixture plots using STRUCTURE for *K* = 2 and *K* = 3 clusters. (c) Spatial autocorrelation plot between spatial distance and genetic relatedness. Moran's I autocorrelation coefficient (*r*) for different distance classes (m). Red line = raw values. Blue lines = permutations. Grey ribbon = 95% CI of permutations. (d) The adult distribution in genetic clusters relative to depth (bathymetry map from Hedley (2012)).

Following PCA, adults were clustered into two main groups (herein Group 1 and Group 2), with colony 12 identified as an ‘outlier’ group owing to the limited samples (Group 3) (Figure 3a). STRUCTURE analysis initially indicated the presence of only two genetic clusters (*K* = 2). However, we also present the population structure at *K* = 3 to account for Group 3, as it's unique genetic signature might not be fully captured when restricted to two ancestral populations (Figure 3a,b). Interestingly, colony 12 showed substantial membership from a genetic group that is distinct from Groups 1 and 2 (Figure 3b). Removal of colony 12 in the population structure or paternity analyses did not affect the main results, and consequently, the colony was retained in the analysis to represent the full morphological range of the species observed in the experiment. There were no clear morphological features that could separate the two main groups by visual inspection (Figure S4). The inbreeding coefficient (*F*) values had a median of 0.499, indicating a high probability of inbreeding within the population.

There was a weak, although significant, effect of linkage disequilibrium assessed via Index of Association (IA) (${\bar{r}}_{d}$ = 0.047, *p* = 0.001) suggesting a small degree of non‐random association of alleles between the loci in the dataset among all individuals. However, after grouping individuals into subpopulations, there were no significant effects for either group (Group 1: ${\bar{r}}_{d}$ = 0.002, *p* = 0.590; Group 2: 0.011, *p* = 0.859). Further analysis revealed that each of the 18 adult individuals possesses a unique multilocus genotype (MLG), indicating an absence of direct clonality (fragments) in the adult dataset.

Spatial autocorrelation analysis, on the basis of a permutation test, revealed a significant pattern of genetic differentiation at the largest spatial scales. The autocorrelation coefficient (*r*) became negative at distances greater than ~90 m, and the value at the furthest distance class (127.6 m) was statistically significant (*r* = −0.39, *p* = 0.017). This indicates that individuals separated by these distances were genetically more dissimilar than expected by chance (Figure 3c). Similarly, genetic groupings appeared spatially separated along the reef slope, but these trends were independent of depth (*t* = 0.898, *p* = 0.394) (Figure 3d).

## 4 Discussion

Despite the wide range of adult colony densities within our patch, outcrossing in *P. daedalea* was absent along this section of reef. This was driven by a confluence of factors: (i) corals spawning asynchronously across months, characterised by a split spawning event where only approximately one in four colonies were gravid across the reef slope, (ii) asynchronous spawning across many nights, further contributing to a fragmented reproductive pattern, and (iii) substantial asynchrony within the same night. This pronounced asynchrony across multiple temporal scales ultimately implies that a relatively limited number of gametes encountered sufficient sperm concentrations necessary for successful outcrossed fertilisation. This was corroborated by the low fertilisation success observed in our collected samples, with no evidence of outcrossed embryos. However, given our limited sample size, we interpret these results with caution, as the low sample number could influence the detectability of outcrossing. Although our study did not experimentally manipulate colony density, the observed relationship between asynchrony, low gamete encounters and reduced fertilisation success is consistent with an Allee effect, where reproductive success declines owing to insufficient gamete availability. Recent studies using similar methods, where fertilisation was tracked from contained eggs sourced from natural and manipulated adult patches, have reported up to 30% outcrossing success in synchronous, high‐density spawners (Mumby et al. 2024; Ricardo et al. 2024). This comparison reinforces that the low success in our patch was likely driven not simply by adult population density alone but also by the compounding effects of temporal asynchrony on fertilisation.

Unusual spawning patterns within single nights have been documented in *Platygyra* previously (Miller 1994b), and we observed one colony spawning multiple times within a single night. However, ordination‐ and model‐based clustering analyses of individuals into two main genetic groups reduced the spawning variability within nights, indicating these two groups may be partly reproductively isolated through differences in spawning timings, potentially indicative of early‐stage allochronic speciation. Here, we avoid using the term ‘cryptic’ species without further population or reproductive data (Patterson et al. 2006; Ramírez‐Portilla et al. 2022). However, morphological stasis is known to mask ecological divergence (Bongaerts et al. 2021); although our population of sampled *P. daedalea* morphologies all fell within the expected range of morphologies for this species, distinct genetic groupings and spawning patterns are apparent, providing evidence that ecological divergence is occurring.

Caution is needed when comparing species with complex taxonomy across studies; however, the observed intercolonial spacing and density of gravid *P. daedalea* colonies were substantially lower than total adult densities reported in previous studies. Even without considering the increased spacing resulting from two genetic groups, the mean intercolonial distance of gravid colonies was high at 7.7 m. These spacings are markedly greater than the 1.2 m distances historically reported for this species on the northern Heron reef flat (Endean et al. 1997). Moreover, gravid colony densities were found to be 71‐fold lower than historic densities on the northern Heron reef flat and 8.4–19.5 times lower than at nearby One Tree Island reef flat (Miller and Ayre 2008). These discrepancies likely reflect both the partial cohort involved in a split spawning event and habitat differences, as our study site was located on the reef slope, where spur and groove formations likely reduced the availability of continuous habitat compared to the reef flat. Contrary to the regular spacing of merulinids observed by Endean et al. (1997), we found a tendency towards spatial clustering in gravid *P. daedalea* colonies. This clustering may be attributed to the spur and groove formations in this section of reef, which likely constrains the availability of suitable habitat space compared to densities reported on the reef flat.

Although self‐fertilisation in many broadcast‐spawning corals is typically low, elevated levels of selfing have been documented in *Platygyra* and other genera (Miller 1994b; Willis et al. 1997). In laboratory settings, the capacity for self‐fertilisation is likely overestimated owing to artificially high sperm concentrations, prolonged egg‐sperm contact times and the absence of competing conspecific sperm. In this study, we found that the few embryos in our samples were genetically close to their maternal parent and exhibited higher homozygous alleles, indicating selfing despite the dilutive conditions and possible sperm competition present in the field. Although our field controls detected no evidence of self‐fertilisation, laboratory controls—where eggs were exposed to saturating self‐sperm concentrations for extended periods—revealed a delayed yet substantial level of fertilisation success. Initial fertilisation was minimal at 3 h but significantly increased by 6‐h, coinciding with the onset of primary cell cleavage, usually observed within 90 to 120 min following spawning. The two‐cell stage observed at 6 h suggests fertilisation instead occurred at approximately 4‐ to 5‐h post‐spawning. Although we cannot disregard the likelihood that these embryos resulted from gynogenetic parthenogenesis, which allows some level of genetic recombination, this phenomenon has not been described in this species and we did not observe parthenogenesis in our laboratory experiments. Therefore, we consider parthenogenesis unlikely. The absence of self‐fertilisation in field controls, on the basis of our observations, suggests that perhaps not all individuals possess the capacity to self.

Delayed self‐fertilisation has been documented in several coral species in environments where conspecific sperm are low or absent (Heyward and Babcock 1986; Willis et al. 1997; Isomura et al. 2016) and combines the advantages of outcrossing and selfing, i.e., a bet‐hedging strategy to produce progeny if a preferred strategy fails (Goodwillie and Weber 2018). The mechanism for delayed selfing is poorly studied in marine invertebrates compared with higher plants (Kalisz et al. 1999; Rea and Nasrallah 2008), but generally results from the relaxation of the self‐incompatibility (SI) system in the gametes as they age (Goodwillie and Weber 2018). In ascidians, gene expression in the egg's vitelline coat, as well as the use of self‐incompatibility molecules have been proposed as a mechanism for self/non‐self‐recognition (Saito and Sawada 2022). Additionally, we observed high inbreeding coefficients in the adult population, which has been linked with an organism's ability to self (Olsen et al. 2020), and may indicate that selfing plays an influential role in shaping the genetic structure of this population.

Contrary to the findings of Miller and Ayre (2008), we did not observe clonal individuals within our transect, even among those in close proximity (e.g., Adults 14 and 15). However, inbreeding coefficients were high and inbreeding has been suggested to be common in marine invertebrates, even those that disperse gametes and randomly mate (Olsen et al. 2020). The spatial autocorrelation analysis revealed a negative, marginally significant, trend between intercolonial distance and genetic relatedness across a fine‐spatial scale. Interestingly, this trend across fine scales has been observed before, even when clones are accounted for (Miller and Ayre 2008; Dubé et al. 2020). Here, negative relatedness values become more pronounced at intercolonial distances of approximately 90 m. This pattern, typical of brooding species whose larvae often settle near the parent colony (Prata et al. 2024), was highly unexpected in broadcast spawners where localised stock‐recruitment relationships are less established in open populations (Doropoulos et al. 2015), but see Ayre and Hughes (2000), Japaud et al. (2019) and Dubé et al. (2020). However, mobile bodies resembling larvae have been visually observed within polyps of a *Platygyra* sp. (R. Babcock pers. comm.), indicating some species in this genus may be undescribed facultative brooders.

An alternative hypothesis to explain this spatial‐genetic correlation is that larvae may preferentially settle near conspecific adult groups. Although this hypothesis contrasts with some existing observations of Janzen‐Connell effects (Janzen 1970), a theory that has been extensively studied in terrestrial systems, these effects have only recently been explored in marine invertebrates at small spatial scales of metres and may require further examination at scales of tens to hundreds of metres (Johnson et al. 2012; Marhaver et al. 2013; Sims et al. 2021). Additionally, the possibility of larvae settling as sibling groups through collective dispersal, although less plausible, could explain the observed spatial patterns (see Eldon et al. (2016) for a review). This scenario depends on cross‐fertilisation among nearby spawning adults (Levitan et al. 2004; Ricardo et al. 2024), followed by the settlement of siblings in close proximity to one another. Despite short larval competency times of *P. daedalea* (Miller and Mundy 2003) and that drifter releases close to the study site indicated that larvae may be retained locally and possibly within the Heron–Wistari channel, divergence of mesh container trajectories even within a few hours of spawning indicates this hypothesis is unlikely. Few studies have assessed siblingship patterns in corals, and further work is needed to assess if such patterns are common or play a meaningful role in shaping spatial‐genetic structures (Puill‐Stephan et al. 2009; Dubé et al. 2020).

*Platygyra* lacks distinct morphological and reproductive traits that allow clear delineation into species i.e., a syngameon. Although the majority of the *P. daedalea* colonies were identified as the Classic PDC *P. daedalea* morphotype (Miller 1994a), clear morphological differences were observed even within this morphotype (Figure S4). Nonetheless, we found no consistent morphological differences corresponding to the genetic partitioning between the two main groups. Colony 12 was a distinct morphological variant (‘fat morph’; K. Miller pers. comm.), but there was only one individual within our transect and therefore we could not compare this colony against other groups. Although some morphotypes within *P. daedalea* have shown reduced cross‐fertilisation success (Miller 1994b), this is unlikely to be the cause of the absence of outcrossing in our experiment because almost all corals in our transect appeared to be the same morph. The genetic clustering we observed contrasted that observed in Miller and Benzie (1997), who observed little genetic differentiation in the genus *Platygyra*, but was more closely consistent with Miller and Ayre (2008) who found some weak genetic subdivision at small spatial scales. The contrast in findings may be owing to true differences in fine‐scale population structure between the studies, but may also be a result of methodological differences i.e., SNP genotyping used here typically resolves greater levels of grouping than observed in microsatellites (Zimmerman et al. 2020; Pérez‐González et al. 2023).

Our results show that limited outcrossing can occur in *Platygyra* during split spawning events when the density of reproductively active colonies is substantially lower and distances between spawning colonies are greater. Reproductive plasticity allowing delayed self‐fertilisation may provide minor reproductive assurance by producing a limited number of offspring when sexual outcrossing fails during low‐density spawning events. However, the predominance of this reproductive mode under typical, higher‐density spawning conditions is unlikely. Patterns of low‐to‐moderate genetic differentiation observed in this species are consistent with ongoing outcrossing and gene flow (Miller and Benzie 1997; Miller and Ayre 2008), suggesting that self‐fertilisation may be more restricted to conditions of low colony density or spawning asynchrony. Although clones were not observed in the samples or transect, as has been observed elsewhere (Babcock 1991; Miller and Ayre 2008), there was some evidence of inbreeding or asexual reproduction in the adult population data. Together, these findings suggest that, without an unidentified reproductive mechanism or hydrodynamic convergence zones, minor spawning events on typical reef tracts may lead to substantial gamete loss—a common feature even in high‐density slicks (Hodgson 1988; Simpson et al. 1993). Even under optimal laboratory conditions, it appears species of *Platygyra* rarely achieves high levels of fertilisation (Miller and Babcock 1997). The contribution of outcrossed larvae may be largely restricted to areas of higher colony densities, both at the reef scale and across the metapopulation, as well as in less uniform reef sections where localised eddies or hydrodynamic retention zones facilitate gamete encounter. In these contexts, certain dense or favourably situated populations may disproportionately contribute, indicating that effective spawning densities and physical conditions must exceed those observed here. Restoration efforts aimed at enhancing effective reproduction on reefs should consider adult densities but also reproductive modes, spawning synchrony and inherent population structure to maximise their effectiveness. Moreover, density‐dependent processes on reefs, starting with the reproduction of mass spawners that rely on external fertilisation, need to be considered in model simulations of reef dynamics. It is highly likely that most simulation modelling efforts to date have not considered such processes and have thus overestimated larval production during recovery phases.

## Author Contributions

**Gerard F. Ricardo:** conceptualization (equal), formal analysis (lead), investigation (lead), methodology (lead), visualization (lead), writing – original draft (lead), writing – review and editing (lead). **Christopher Doropoulos:** conceptualization (equal), funding acquisition (equal), investigation (supporting), methodology (equal), supervision (equal), writing – review and editing (lead). **Russell C. Babcock:** investigation (lead), methodology (equal), writing – review and editing (supporting). **Arne A. S. Adam:** formal analysis (supporting), writing – review and editing (supporting). **Elizabeth Buccheri:** investigation (supporting), writing – review and editing (supporting). **Natalia Robledo:** investigation (lead), writing – review and editing (supporting). **Julian Uribe‐Palomino:** investigation (supporting), writing – review and editing (supporting). **Peter J. Mumby:** conceptualization (equal), funding acquisition (equal), investigation (lead), methodology (equal), supervision (equal), writing – review and editing (lead).

## Conflicts of Interest

The authors declare no conflicts of interest.

## Supporting information


Appendix S1.

## Data Availability

Data and code underlying this manuscript currently are archived and publicly available at https://github.com/gerard‐ricardo/Heron_Allee. The datasets used for this study are deposited in the CSIRO Data Access Portal https://data.csiro.au/.
