# Supplementary material for: Spawning Asynchrony and Mixed Reproductive Strategies in a Common Mass Spawning Coral
Source: Ecol Evol. 2025 Jun 26;15(7):e71654. doi: 10.1002/ece3.71654 (PMC12202782; doi:10.1002/ece3.71654)
Supplement: Supplementary file 1 — Appendix S1. [file ECE3-15-e71654-s001.docx]

# Appendix

Text S1. *Acropora kenti* (n = 11) adult colonies were collected from Orpheus (18.6194°S, 146.4859°E) and Pelorus Island (18.5374°S, 146.4934°E) reefs in Nov 2022. Following spawning, a larval culture was created between two known parents. Samples of each colony and three 42-hour-old larvae from the culture were placed in 100% ethanol solution, and stored at -20°C. These samples were sent for SNP sequencing (0.25 mln reads) and basic filtering at DarT P/L as described above. SNP data underwent further filtering that reduced the total number of usable loci to 257, which is typically sufficient to predict parent-offspring pairs in SNP data (Anderson & Garza 2006).

Following assignment of one of the known parents as the dam (egg donor colonies), the following settings were used: simulated offspring = 20,000, candidate sires (sperm donors) = 10, proportion of colonies sampled = 0.99, proportion of loci typed = 0.99, proportion of loci mistyped = 0.01, minimum typed loci = 128. All larvae were assigned correctly to their known sperm donor as the most likely candidate parent (Fig. S2). Although none of the assignments indicated high levels of confidence—likely due to the low-density sequencing used for this preliminary analysis—our tests successfully assigned a candidate sperm donor.

Table S1. Individual times of spawning of *Platygyra* *daedalea* across spawning nights. DAFM = Days After the Full Moon. Brackets denote the number of colonies spawning at the timepoint. Times are standardised to last light (6:58 pm). Note: some colonies spawned on multiple days and twice on the same night.

| Morphotype | 5 DAFM | 6 DAFM | 7 DAFM |
| --- | --- | --- | --- |
| PDC (Classic) | 0:52 (3), >2:52 (1) | -0:12 (2), -0:06 (2), 0:50 (1) | >0:38 (4) |
| PH (Hillocky) | – | -0:16 (1) | – |
| PDSE (Encrusting) | – | – | >0:38 (1) |

Table. S2. CERVUS output for paternity assignments. Two replicate samples of candidate sperm donor colonies were provided in the analysis.

| Offspring ID | Egg donor ID | Candidate sperm donor ID | Trio LOD score | Trio confidence |
| --- | --- | --- | --- | --- |
| pd13_l_14_11 | pd13_a_1 | pd13_a_2 | 9.08E+01 | * |
| pd13_l_14_12 | pd13_a_1 | pd13_a_2 | 8.69E+01 | * |
| pd13_l_14_13 | pd13_a_1 | pd13_a_2 | 1.03E+02 | * |
| pd13_l_14_14 | pd13_a_1 | pd13_a_2 | 7.41E+01 | * |
| pd13_l_14_16 | pd13_a_1 | pd13_a_1 | 2.25E+00 | * |
| pd13_l_14_3 | pd13_a_1 | pd13_a_2 | 1.04E+02 | * |
| pd13_l_14_4 | pd13_a_1 | pd13_a_2 | 1.06E+02 | * |
| pd13_l_14_5 | pd13_a_1 | pd13_a_2 | 1.02E+02 | * |
| pd13_l_14_6 | pd13_a_1 | pd13_a_2 | 8.47E+01 | * |
| pd13_l_14_7 | pd13_a_1 | pd13_a_2 | 1.01E+02 | * |
| pd13_l_14_8 | pd13_a_1 | pd13_a_2 | 1.01E+02 | * |
| pd14_l_14_1 | pd14_a_1 | pd14_a_1 | 1.12E+02 | * |
| pd14_l_14_2 | pd14_a_1 | pd14_a_1 | 1.23E+02 | * |
| pd14_l_14_3 | pd14_a_1 | pd14_a_1 | 1.22E+02 | * |
| pd14_l_14_4 | pd14_a_1 | pd14_a_1 | 1.32E+02 | * |
| pd15_l_13_1 | pd15_a_1 | pd15_a_2 | 1.05E+02 | * |
| pd15_l_14_1 | pd15_a_1 | pd15_a_2 | 8.94E+01 | * |
| pd15_l_14_2 | pd15_a_1 | pd15_a_1 | 9.34E+01 | * |
| pd15_l_14_3 | pd15_a_1 | pd15_a_2 | 9.84E+01 | * |
| pd15_l_14_4 | pd15_a_1 | pd15_a_2 | 9.97E+01 | * |
| pd15_l_14_5 | pd15_a_1 | pd15_a_1 | 1.00E+02 | * |
| pd15_l_14_7 | pd15_a_1 | pd15_a_2 | 9.59E+01 | * |
| pd15_l_14_9 | pd15_a_1 | pd15_a_1 | 8.85E+01 | * |
| pd9_l_14_2 | pd9_a_1 | pd9_a_1 | 1.23E+02 | * |
| pd9_l_14_4 | pd9_a_1 | pd9_a_1 | 1.30E+02 | * |
| pd9_l_14_5 | pd9_a_1 | pd9_a_2 | 1.19E+02 | * |
| pd9_l_14_6 | pd9_a_1 | pd9_a_1 | 1.12E+02 | * |
| pd9_l_14_7 | pd9_a_1 | pd9_a_1 | 1.17E+02 | * |

Table. S3. COLONY output for paternity assignments Two replicate samples of candidate sperm-donor colonies were provided in the analysis.

| OffspringID | Inferred_sperm_donor | Inferred_egg_donor | Probability |
| --- | --- | --- | --- |
| pd13_l_14_3 | pd13_a_1 | pd13_a_1 | 0.9999 |
| pd13_l_14_4 | pd13_a_1 | pd13_a_1 | 1 |
| pd13_l_14_5 | pd13_a_1 | pd13_a_1 | 1 |
| pd13_l_14_6 | pd13_a_1 | pd13_a_1 | 0.9999 |
| pd13_l_14_7 | pd13_a_1 | pd13_a_1 | 0.9999 |
| pd13_l_14_8 | pd13_a_1 | pd13_a_1 | 0.9999 |
| pd13_l_14_11 | pd13_a_1 | pd13_a_1 | 1 |
| pd13_l_14_12 | pd13_a_1 | pd13_a_1 | 1 |
| pd13_l_14_13 | pd13_a_1 | pd13_a_1 | 1 |
| pd13_l_14_14 | pd13_a_1 | pd13_a_1 | 0.9999 |
| pd14_l_14_1 | pd14_a_1 | pd14_a_1 | 1 |
| pd14_l_14_2 | pd14_a_1 | pd14_a_1 | 0.9969 |
| pd14_l_14_3 | pd14_a_1 | pd14_a_1 | 0.9991 |
| pd14_l_14_4 | pd14_a_1 | pd14_a_1 | 1 |
| pd15_l_13_1 | pd15_a_1 | pd15_a_1 | 0.9135 |
| pd15_l_14_1 | pd15_a_1 | pd15_a_1 | 0.9143 |
| pd15_l_14_2 | pd15_a_1 | pd15_a_1 | 0.9075 |
| pd15_l_14_3 | pd15_a_1 | pd15_a_1 | 0.9117 |
| pd15_l_14_4 | pd15_a_1 | pd15_a_1 | 0.9124 |
| pd15_l_14_5 | pd15_a_1 | pd15_a_1 | 0.9128 |
| pd15_l_14_7 | pd15_a_1 | pd15_a_1 | 0.9139 |
| pd15_l_14_9 | pd15_a_1 | pd15_a_1 | 0.9134 |
| pd9_l_14_2 | pd9_a_1 | pd9_a_1 | 0.7497 |
| pd9_l_14_4 | pd9_a_1 | pd9_a_1 | 0.7944 |
| pd9_l_14_5 | pd9_a_1 | pd9_a_1 | 0.7998 |
| pd9_l_14_6 | pd9_a_1 | pd9_a_1 | 0.5071 |
| pd9_l_14_7 | pd9_a_1 | pd9_a_1 | 0.9683 |


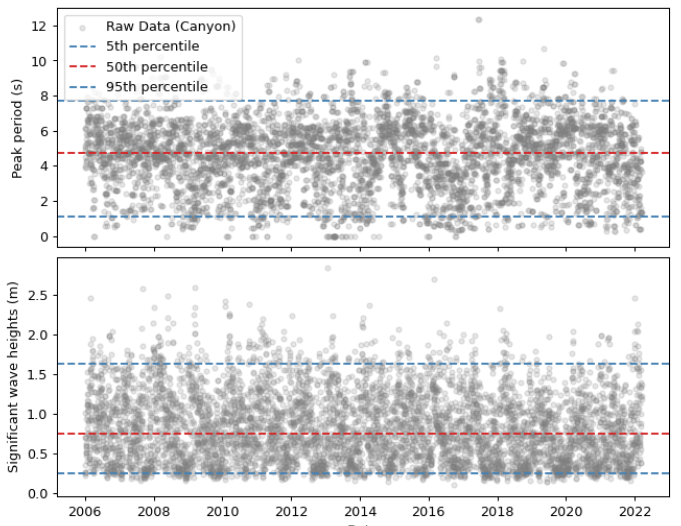


Fig. S1. Modelled wave time series data from 2005–2022 at the field site (Canyons, Heron Island) including peak period and significant wave heights (Callaghan 2023).


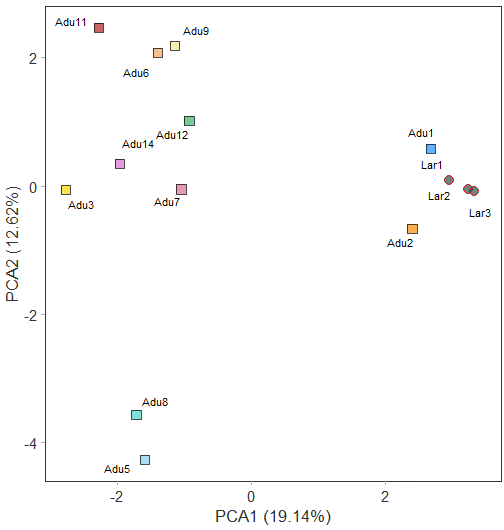


Fig. S2. Principal Components Analysis (PCA) of adult candidate parents (n = 11) and larvae (n = 3) of *Acropora kenti*. Larvae 1 to 3 belonged to known parents, Adults 1 and 2.


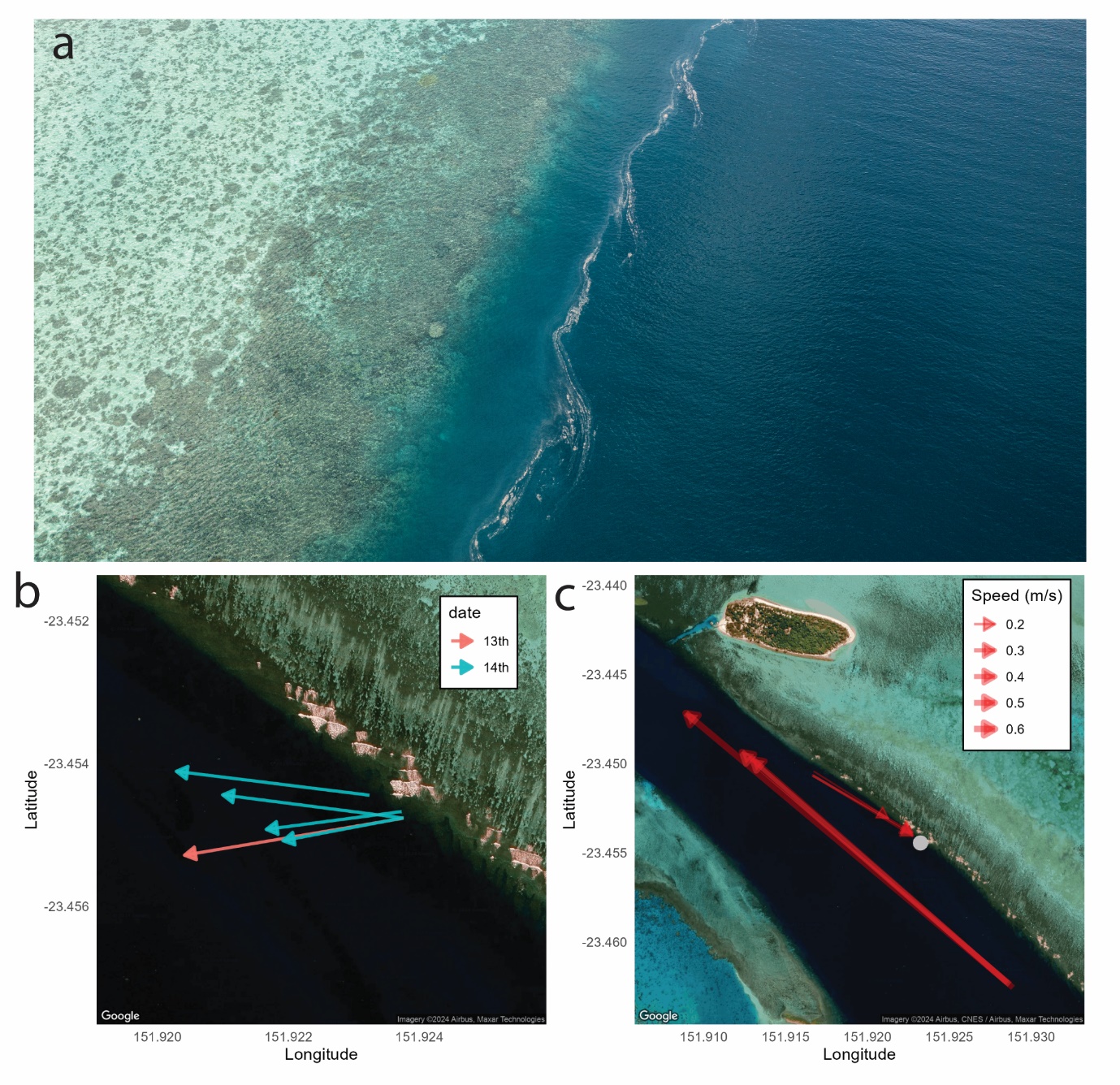


Fig. S3. Hydrodynamics of the field site. (a) A topographically controlled front indicated by a coral spawn slick on the western side of Heron reef in 2021 on the day following spawning (© CSIRO. Credit: Nick Thake 2021). (b) Movement of the mesh containers following release for each spawning night. (c) Movement and speed of drogues released within the Heron-Wistari channel. The grey point denotes the field site.


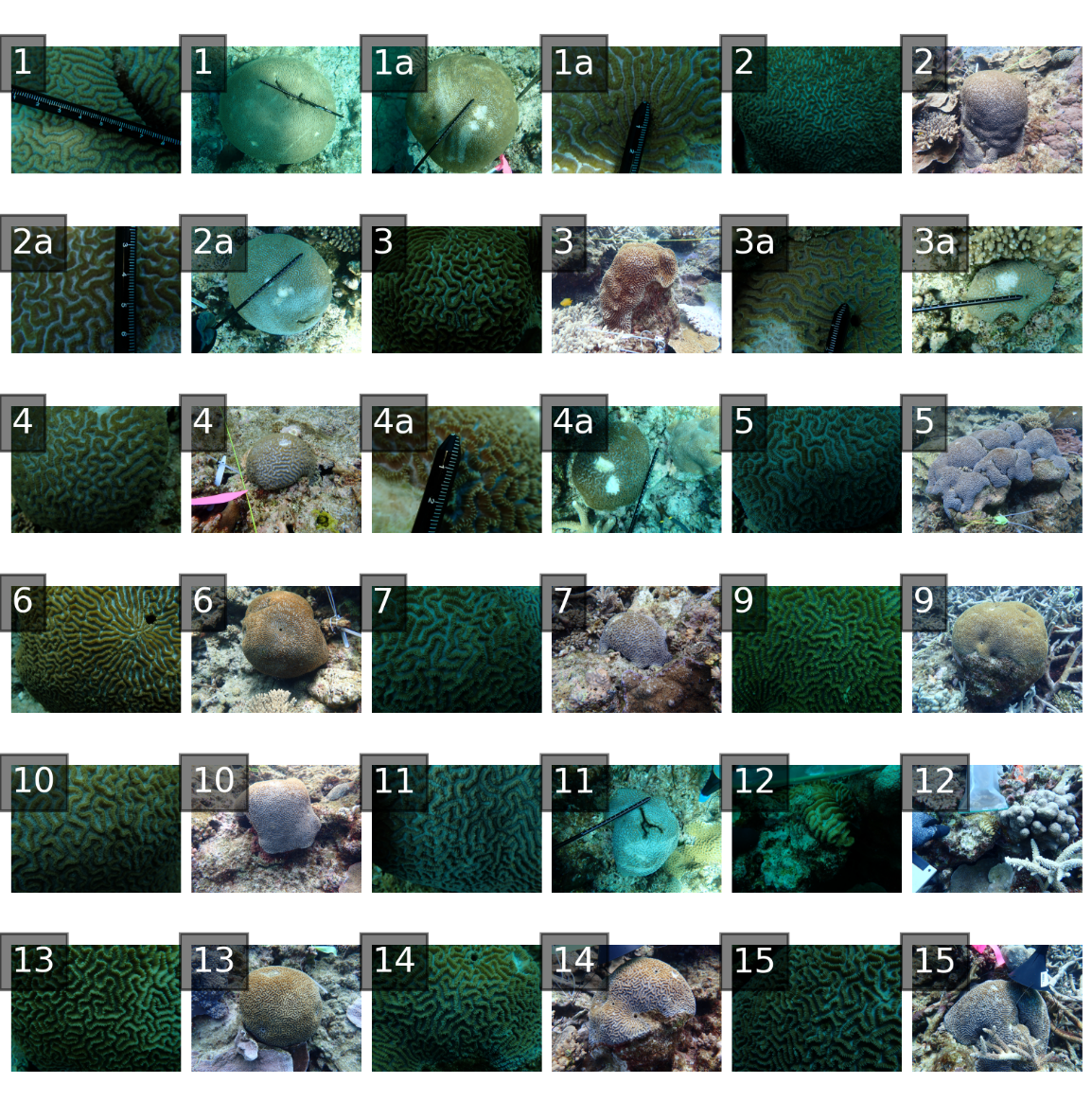


Fig. S4. Paired images (zoomed-in and zoomed-out) of adult *Platygyra daedalea* colonies tagged within the site. Each colony is shown with a unique ID number, corresponding to the colony number used throughout the study. Higher resolution images are available at <https://doi.org/10.6084/m9.figshare.27934917>.
